# The Role of Coaches in a School Leader Preparation Program: “The Cooperative Triad”

**DOI:** 10.3390/bs16050798

**Published:** 2026-05-17

**Authors:** Lacey E. Seaton, Samantha T. Hope, Suhani S. Vakil

**Affiliations:** 1 Department of Educational Leadership, Policy and Human Development, North Carolina State University, Raleigh, NC 27695, USA; 2 School of Education, Virginia Commonwealth University, Richmond, VA 23220, USA; hopest@vcu.edu (S.T.H.); vakilss@vcu.edu (S.S.V.)

**Keywords:** internship coaches, leadership coaching, school leader internship, principal preparation, cooperative triad

## Abstract

As aspiring school leaders or interns complete their graduate program and year-long school-based internship in an urban school setting, they receive support from an on-site school leader mentor and an external coach. This trio, coined the cooperative triad, fosters leadership development through a collaborative approach. When alumni begin their career as a school leader, they continue receiving support from the leadership program as well as an external coach. Because of their important role, this study sought to understand how coaches conceptualized their role, what influenced their ability to carry out the role, and their influence on the internship and alumni experience. Utilizing a qualitative case study design, researchers engaged in data collection and analysis across interviews with seven coaches, seven interns/alumni, and five mentors. Findings highlight the importance of creating a coaching structure while remaining flexible, building trust and community with participants, and continually developing coaches in their practice. Further, the cooperative triad model demonstrates the potential for positive school-wide impact, as the coach supports both aspiring and practicing leaders.

## 1. Introduction

Modern-day educators face many challenges in their day-to-day lives; school districts and states enforce specific requirements that must be balanced against the needs of the students. School leaders have additional responsibilities of overseeing their building, meeting the needs of their staff, and ensuring that school-specific goals are met. With these challenges in mind, the preparation of new leaders requires strategic development in all skill areas as part of their program. As such, this study explores one university-based principal preparation pathway, the Eastern School Leader Preparation Program (ESLP), a program that augmented the traditional year-long school-based administrative internship with rigorous tasks and support structures. When ESLP aspiring school leaders, referred to as interns, complete their graduate program and take on more leadership responsibility in their internship placements, they receive support from a school leader mentor based in the building as well as an external coach, creating a cooperative triad. As alumni begin their careers as school leaders, they continue receiving support from the leadership program, which includes an external coach. Because coaches play such an important role in the leadership development program, this study sought to understand how coaches conceptualized their role, what influenced their ability to carry out the role, and their influence on the internship and alumni experience.

## 2. Literature Review

The practice of leadership coaching is used across multiple professions; athletes, corporate executives, and educators all experience some form of coaching during their careers. While school leader preparation does not follow one model, in some programs, coaches are provided to support the aspiring school leader’s development. Although there is no singular definition of coaching, common themes across disciplines include growth and improvement of the individual being coached (Watling & LaDonna, 2019) by, in part, achieving agreed-upon goals (James-Ward, 2013; Salavert, 2015; Su-Keene & DeMatthews, 2022). The International Coaching Federation (2025) defines coaching as “partnering with clients in a thought-provoking and creative process that inspires them to be their best personal and professional selves.” Furthermore, Aguilar (2017) describes a coach as “a teacher, a facilitator of someone else’s learning” (p. 2). In the context of supporting aspiring school leaders, there is an important distinction between a coach and a mentor, as some programs utilize both. The coach is often an external support (Snodgrass Rangel et al., 2024), typically an experienced principal, while the mentor is the principal in the school building that the aspiring leader is shadowing. Some researchers view coaching as a one-on-one activity between the coach and the individual being coached (e.g., van Nieuwerburgh et al., 2020), while others have examined it from a triad perspective through which coaches hold conversations with both the individual being coached and their mentor, often the coachee’s on-site supervisor (e.g., Cosner et al., 2018). In the triad model, Cosner and De Voto (2023) introduce the concept of the coach as a “broker” (p. 10), which helps them serve in an intermediary and practical capacity. In this role, the coach can help the aspiring school leader and their mentor by connecting them with resources, providing mediation if needed, and engaging with both parties in leadership activities.

Leadership coaching is becoming commonly understood as a relational, reflective process centered on growth and development (Boon, 2022; Newcomb et al., 2026). Coaches are generally not seen as evaluators, but rather as someone who can provide feedback and guidance to build leadership capacity (Kappler-Hewitt et al., 2020; Taylor, 2008). Integral to this relational, reflective process is active listening and purposeful questioning (Augustine-Shaw & Reilly, 2017; Patrick et al., 2021; Salavert, 2015). Stevenson (2017) builds upon the importance of strategic questioning, explaining that coaches should assess when those they support “need concrete information, when they need ways to think about problems, and when they will most benefit from questions that challenge their assumptions” (p. 35). Given the personal nature of coaching, trust is an essential component in developing a safe relationship where the aspiring leader can authentically receive and apply feedback (Snodgrass Rangel et al., 2024; Stevenson, 2017). Several factors can influence the development of trust, including “differences in gender and race, prior professional experiences, the presence of pre-existing professional relationships, and the complexity of the district’s organizational structure” (Lochmiller, 2024, p. 287). Developing trust and providing effective communication are key features of quality coaching (van Nieuwerburgh et al., 2020; Wise & Hammack, 2011).

Coaching extends beyond building a strong relational foundation to intentionally advance school leaders’ instructional practice in ways that analyze and improve student achievement (Almager et al., 2021; Collins et al., 2025; Master et al., 2022). Moreover, numerous studies cited by Darling-Hammond et al. (2022) show that school leaders who receive mentoring and/or leadership coaching support demonstrate stronger instructional leadership, which in turn results in improved teaching practices and student achievement outcomes. Although instructional development and feedback are common components when coaching sitting principals, these concepts can be applied to aspiring school leaders. For example, Gray (2018) established the Leadership-Focused Coaching framework, which is designed to develop aspiring and novice school leaders by highlighting instructional leadership. The benefits of coaching extend beyond instructional leadership. Understanding coaching outcomes, as well as mechanisms for selecting and supporting coaches, helps create the foundation for this study.

### 2.1. Selection & Support

As with defining coaching, there are different approaches to the selection, preparation, and support of coaches. Quality preparation programs carefully select their coaches, with preference given to those who are external to the school district in which the aspiring school leader and their mentor are employed (Kappler-Hewitt et al., 2020). Having an external coach can provide the intern with a trusting, non-evaluative person that they can reach out to for help and advice (Fusarelli & Fusarelli, 2023). An external coach provides a sense of security and comfort to a coachee who, when experiencing challenges, often needs space to be vulnerable in order to unpack barriers and establish a plan to progress forward. Conversely, Wise and Cavazos (2017) assert that coaches are ideally individuals from within the same district as the interns they support. However, not all researchers believe it matters whether or not the coach is external to the school district. For example, Salavert (2015) argues that the skillset of the coach is a more important factor and explains that to be successful in the role, a coach should have extensive leadership experience, be an effective communicator, and have a proven track record of building teams. Identifying effective selection protocols and coaching structures can give programs a starting point for determining who to choose for the role as well as developing goals for training and ongoing support.

Numerous studies emphasize the importance of consistent and ongoing coaching between the intern and their coach (Cosner & De Voto, 2023; Kappler-Hewitt et al., 2020; Snodgrass Rangel et al., 2024). Coaching structures such as face-to-face and non-face-to-face check-ins serve to provide a consistent, confidential space for the coach and aspirant to communicate, reflect, monitor progress, and share feedback. Geographic proximity should be taken into account when selecting coaches to ensure that they can hold face-to-face coaching sessions (Cosner et al., 2018; Kappler-Hewitt et al., 2020; Master et al., 2022; Wise & Cavazos, 2017). Being able to regularly visit the intern in their clinical placement enables the coach to observe their leadership in action and to hold triad meetings (Cosner et al., 2018). For example, Wise and Cavazos (2017) recommend that formal coaching sessions occur at least once a month, lasting about one to two hours each. Researchers have also encouraged phone calls and text messages to be used between in-person meetings (Cosner et al., 2018; Kappler-Hewitt et al., 2020). Holding regular face-to-face meetings and being in constant contact with the intern strengthens the relationships (Snodgrass Rangel et al., 2024) and allows the coach to make adjustments and provide resources as needed.

While limited research exists regarding the structures and supports needed to train and develop coaches (Collins et al., 2025), some researchers have provided recommendations. For example, creating a professional community where coaches share resources and collaborate with one another offers informal development to coaches working with aspiring leaders (Crow & Whiteman, 2016; Fusarelli & Militello, 2012) as well as practicing principals (Bloom et al., 2003). Likewise, participating in formal training can help coaches develop role-specific skills and strategies, including the ability to troubleshoot internship challenges before they occur (Cosner & De Voto, 2023). Coaches should consistently engage with one another in community, collaborating and building skills in order to continually strengthen their individual practice and their collective impact (Huggins et al., 2021). Leadership preparation programs should make a concerted effort to better understand coaching structures and supports that enhance quality coaching and efficacy (Collins et al., 2025). Studies focused on designing effective coaching will not only contribute to the research field, but will also help preparation programs in their implementation of the coaching role.

### 2.2. The Influence of Coaches

Leadership coaching is associated with numerous positive outcomes for aspiring school leaders, such as self-awareness, skill development, and a sense of community (Almager et al., 2021; Collins et al., 2025; Halliwell et al., 2023). Coaches bridge the gap between the program and internship experience, resolving relational or programmatic issues that arise at the internship site (Cosner & De Voto, 2023; Drake et al., 2023). Even when programs provide clear structure and requirements through proactive planning, gaining access to adequate experiences or navigating complex challenges at times necessitates the coach facilitating reactive problem-solving (Crow & Whiteman, 2016). Creating environments in which the mentor, coach, and coachee work in collaboration to provide authentic leadership opportunities coupled with collective reflection enhances the leadership skills of both the intern and the mentor (Snodgrass Rangel et al., 2024). Similarly, Cosner et al. (2018) and Cosner and De Voto (2023) found that the coach’s role not only benefits the interpersonal relationship of the coachee and mentor but also fosters the aspirant’s leadership knowledge and skills.

One-on-one leadership coaching provides a safe, reflective space that increases interns’ confidence and leadership effectiveness (van Nieuwerburgh et al., 2020). Coaching can also increase new leaders’ sense of belonging (Shoho et al., 2012). Coaching positively contributes to the development of aspiring school leaders through modeling and discussing key leadership responsibilities such as implementing research-based leadership practices and making informed decisions (Gray, 2018). Another valuable outcome of coaching is the human connection it fosters, providing increased opportunities for socialization and professional networking (Roberts & Gonzalez, 2023). This can ultimately help reduce the isolation school leaders often feel, leading to greater retention (Rogers & VanGronigen, 2023). While focused on early-career principals, Rogers and VanGronigen’s work is relevant to those in a novice stage of their leadership who benefit from coaching and support. As such, extending coaching beyond the internship year helps the continual development of new school leaders (Boon, 2022; Fusarelli & Fusarelli, 2023; Kappler-Hewitt et al., 2020).

### 2.3. Theoretical Framework

Developed in the field of sociology, social capital theory explains that relationships between people provide access to information that can lead to growth and productivity. Coleman (1988) explained that social capital makes “possible the achievement of certain ends that in its absence would not be possible” (p. 98). Coaching and mentoring can increase an individual’s social capital, leading to positive outcomes that might not otherwise have been realized. Social capital through coaching and mentoring has helped new personnel become acclimated to the workplace culture more quickly (Uen et al., 2018), increased undergraduate students’ self-efficacy (Aikens et al., 2016), improved the career growth of graduate students (Dolan & Willson, 2019), and furthered the development of aspiring school leaders (Cosner et al., 2018, Rogers & VanGronigen, 2023). Aikens et al. (2016) noted that increasing an individual’s social network increases their social capital, in turn providing greater access to ideas and resources; the researchers found that increased social capital through mentoring relationships positively influenced undergraduate students in numerous ways, including preparation for a future career and increased scientific thinking. Similarly, Cosner et al. (2018) found that coaches increased the social capital of aspiring school leaders by bringing others into the conversation, such as mentors, to create a more robust coaching network. Research indicates the positive outcomes of increased social capital on individuals receiving coaching and mentoring. It is likely that the same is true for those providing coaching or mentoring; a cooperative coaching triad model may benefit the coach and the mentor as much as it benefits the individual being coached.

## 3. Program Background

Aspiring school leaders (referred to as interns) are selected to participate in ESLP through an application and interview process that carefully explores their leadership motivations, evidence of their educator effectiveness, and their leadership goals. At minimum, applicants must hold a master’s degree in the field of education or a master’s degree that meets the requirements for a teaching position; a cumulative GPA no lower than 3.0; an active teaching license, and at least three years of successful teaching experience. While the program was originally designed as a residency in which participants would be released from their full-time positions, due to funding constraints, interns engaged in a traditional university-based, administrative internship supplemented by rigorous residency tasks. Interns complete a graduate certificate in Educational Leadership while simultaneously implementing what they are learning in a high-need PK-12 urban school environment through a gradual release model. Specific to the traditional university-based, administrative internship model, interns are overseen by a mentor principal and complete at least 320 supervised hours of leadership activities that span grades PK-12, the central office, and outside agencies. Additionally, they complete course-embedded projects, including field-based action research projects.

Residency components provide an added layer of support and development for the intern and mentor principal through an external internship coach, creating a cooperative triad model. This external coach reviews and provides feedback on leadership tasks, holds monthly in-person check-ins, and offers less formal as-needed touchpoints. Interns participate in bi-weekly seminars focused on restorative practices, emotional intelligence, child and adolescent mental health, and instructional coaching, applying their learnings directly in internship settings. Finally, the enhanced internship includes an evaluation process, completed by the intern and their mentor, grounded in state performance standards for practicing principals. This process features in-depth reflections on leadership competencies, goal setting, and pre- and post-assessments of the intern’s leadership performance.

After the internship year, program graduates are paired with a new coach and participate in two years of career coaching. Career coaching supports graduates in achieving their desired leadership roles, thriving in new leadership positions, and enacting transformational change in their schools and districts. Graduates are also supported to create a leadership portfolio highlighting their accomplishments and to track progress in focus areas aligned with the practicing principal standards introduced in their internship year. During this time, program graduates and their coaches meet bi-weekly, with informal check-ins often occurring between meetings.

Internship coaches are a support and guide for both interns and mentors and, as such, must have a minimum of three years of experience as a school leader with a proven track record of fostering a positive school culture and student achievement. To be selected as a coach, candidates submit their resume and engage in a comprehensive interview. Interviews not only explore candidates’ tenure of leadership work, but also probe candidates on their knowledge and skills of performance standards for principals, such as instructional leadership, school climate, and organizational management. Coaches in the program have leadership experience ranging from building-level (e.g., principal) to district-level (e.g., superintendent).

As part of the onboarding and support process, coaches participate in an orientation, including a full day of learning about the program processes and expectations. The ESLP staff hold monthly check-ins with coaches to provide guidance on program expectations, space for collaborative problem-solving conversations, and instruction on how to provide specific, actionable feedback. During the monthly check-ins, coaches also engage in mini professional learning sessions where they learn about topics such as principles of adult learning (Knowles et al., 2005), New Teacher Center’s (n.d.) formative assessment tools and supports, and CASEL’s (n.d.) five constructs of transformative social-emotional learning. These professional learning opportunities help coaches understand how adults learn and prefer to receive feedback, as well as how to deliver bite-sized and actionable feedback. Furthermore, coaches may attend all bi-weekly seminars with interns, but are required to participate in instructional leadership seminars that address topics such as coaching entry points, coaching language and stances, feedback styles and recommendations, and essential instructional tools, including analyzing student learning and the observation cycle.

## 4. Materials and Methods

### 4.1. Research Design & Participants

This paper is part of a larger qualitative case study on leadership preparation focused on the school leader internship. Due to the small size of the program, the researchers used purposeful selection to invite all individuals who had served as a coach, mentor, or intern/alumni to participate in the study in an effort to ensure that the research questions could be answered using multiple viewpoints (Maxwell, 2013). Using the research questions as a guide, interview protocols were developed for each group of participants to capture their experience with coaching. Researchers piloted the coach interview protocol (Creswell & Poth, 2018) with an individual who served in a unique position as a program-school district liaison during the program’s planning year, during which she supported the program’s sole intern while the coaching model was being developed. Invitations to participate in this study were then extended to eight coaches, of which seven agreed to participate; ten interns/alumni, of which seven agreed to participate; and eight mentors, of which five agreed to participate. Semi-structured interviews were conducted between December of 2024 and July of 2025 via Zoom, each approximately one hour in length (Flick, 2018). While the focus of this paper is the program’s coaches specifically, findings were triangulated (Yin, 2016) across interviews from seven interns/alumni and five mentors from the same research project to ensure our themes aligned across all 19 participants. The interviews were audio-recorded and transcribed using Zoom’s built-in transcription feature. The recordings allowed the researchers to verify the accuracy of the transcripts when using direct quotes.

### 4.2. Data Analysis

The data analysis process began immediately after each interview was conducted. In an effort to enhance validity, the same two interviewers conducted each interview, debriefed immediately following each interview, and then analyzed each interview separately. Because of their familiarity with the data, the researchers took a “derived notes” (Yin, 2016, p. 200) approach to analyzing interview data rather than coding interview transcripts. Yin defines derived notes as: “A new set of substantive notes, drawing directly from the original field notes and collected qualitative data, to be used as the basis for analyzing and interpreting the data” (p. 335). Yin further explains that, “The derived notes are used in lieu of any formal coding of the data and therefore represent a noncoding option for analyzing the data” (p. 335). Through this inductive approach, which Yin (2016) defines as a way of letting “the data lead to the emergence of concepts” (p. 100), the researchers developed separate notes for key themes that emerged from the data. Direct interview quotes, researcher impressions, and data from other sources were included in these documents. The research questions guided our consideration for specific focal areas, such as the coach’s role, guiding structures, and their potential influence across program stakeholders. Nevertheless, the use of an inductive approach allowed the researchers to remain open-minded by refraining from using predetermined themes. The data from all 19 participants were reviewed through several rounds of analysis as the researchers worked to understand the thematic development across all sources. As such, the research questions shaped our analysis while the derived notes kept the findings grounded in participant voices.

The researchers met weekly to discuss the data and review the derived notes for each group of participants (i.e., coaches, mentors, and interns/alumni). They developed a list of themes (Saldaña, 2013) found in the data for each group and then created a table to triangulate the data, showing commonalities in themes across the three groups of participants. For example, researchers established the overarching theme of ‘The Structure and Fluidity of Coaching’ through reviewing notes from the coach interviews focused on four topics, listed below, each of which is illustrated by a quote from the data:

- Resident Drives the Conversation: “A free-flowing conversation was emphasized. But at the same time, you also try to keep it structured to where you’re meeting their needs.”
- Scheduling: “The meetings usually last as long as needed. A lot of times it depends on the candidate.”
- Meeting Them Where They Are: “Meet them where they are in a graceful way, and just like you do as a school administrator with teachers, some you have to be a little bit harder on. Some you have to be kinder on.”
- Flexibility & The Triad: “Today was a good example of when the principal just can’t be there. He was dealing with a super noncompliant kid. So he and I will touch base later today, probably by email or phone, whatever he prefers.”

This overarching theme of ‘The Structure and Fluidity of Coaching’ was triangulated with the data related to ‘Flexibility’ from the mentor interview notes, and ‘Coaching Meeting Structure’ and ‘Flexibility in Meeting’ from the intern interview notes. To ensure transparency in our theme development, Table 1 displays the alignment between the findings, data themes, and research questions. By triangulating the data across all participant groups when building a theme, the researchers strengthened the findings by representing various viewpoints across themes as opposed to isolated experiences.

To increase the trustworthiness of our methodological approach, the researchers employed several strategies: the use of data triangulation, reaching consensus among researchers for theme development, and including the researcher’s reflexivity statement below (Creswell & Creswell, 2018). Data triangulation allowed the researchers to confirm common experiences across participants, reducing researcher bias in the findings. Likewise, through discussions about data analysis, the researchers reached consensus on the findings based on the available data. Additionally, the researchers who collected and analyzed the data co-wrote the findings and discussion sections during weekly meetings, strengthening the overall methodology. Through these recurring live writing sessions, the researchers engaged in discourse, revisited the data, and ensured that counter-narratives to themes were included. This study sought to understand the role of the coach in one leadership preparation program. To do so, the following research questions were posed:

1. How is the role of the coach conceptualized across coaches, mentors, interns, and alumni throughout a school leader preparation program?
2. How do coaching structures, practices, and support influence the coach’s ability to carry out the role?
3. How, if at all, does the coach influence the overall internship experience across all stakeholders?
4. How, if at all, does the coach influence the transition to leadership for alumni?

### 4.3. Reflexivity

In an effort to provide transparency regarding the connection between the authors and the research, we provide our reflexivity statements here (Yin, 2016), including how we worked cooperatively to limit biasing the research and ultimately the findings. The first author worked at the program being studied during the time in which the internship was being redesigned to reflect more of a residency-style model. At the time of the research study, this author had shifted employment to a different university to work with a leadership preparation program that is designated as exemplary by UCEA. Her new university employer is also her alma mater, where she completed her full-time leadership residency. As such, the primary author, while having direct knowledge of the ESLP, is also in a position to offer direct critique as a current outsider. The second author serves as a data and evaluation specialist for the program of study. Although a former public school educator, she has not attended a school leadership program nor served as a school leader. She is primarily motivated to study the program in an effort to improve its design and outcomes. The final author has both school- and district-level leadership experience and served as a program lead for the program of study. As such, this author contributed to the research design and literature review but did not participate in the collection and data analysis portion of this research in order to limit their biasing of the participants or the findings. In consideration of our positioning, as a final way to ensure consistent interpretation of the findings, as previously noted, the two primary researchers completed each portion of the analysis in unison to increase validity (Maxwell, 2013).

## 5. Findings

Data were analyzed to address the four research questions regarding conceptualization of the internship coaching role, structures that shape the coach’s work, the coach’s influence on stakeholders during the internship, and the coach’s influence on the transition to leadership for program alumni. The qualitative analyses surfaced overarching themes, including the triad relationship, the importance of both structure and fluidity to coaching sessions, and how coaches were able to grow in their practice. As various stakeholders shared their experiences during the internship and post-graduation, special emphasis was placed on how the triad relationship filled gaps within the internship and supported the mentor in addition to the intern. The social capital coaches brought to the role, coupled with that provided by the mentor, ensured the intern had access to a broad professional network and gained valuable leadership experience. While clear themes emerged amongst the coaches, some experiences were unique to specific coaching triads. Throughout this section, we will contextualize details related to setting and relational dynamics as we describe each theme.

### 5.1. The Role and Influence of the Coach

Throughout the interviews, coaches shared their perspectives of what it means to be a coach. They demonstrated a consistent understanding of program expectations for the coaching position. Similarly, mentor and intern interviews revealed a shared appreciation of the role of coaches. Holistically, coaches emphasized their role as being relational and supportive while simultaneously providing a layer of structure and accountability. Imani noted, “I think the biggest thing about the coach is being able to mentor and support both the [intern] and the school leader mentor.” She elaborated by saying, “Being able to really listen and understand where they’re coming from, but at the same time push where you need to.” Carl provided his understanding of the role as being “a sounding board, a champion, somebody who has lived experience” and he further elaborated that the purpose of the external coach is “to help them [the intern and mentor] think through tough situations.” Lucy echoed Carl’s perspective of the coaching role by describing it as a “combination of a coach, a mentor, an advisor, kind of like an all-round whatever they needed.” She further shared:

I was what I would always call that trusted ear. So if there was something not going right or something didn’t feel right or there was a question. I was a trusted person that would not have a negative implication on their school or job.

Coaches advocated for interns to have experiential learning opportunities, and guided interns and their mentors to identify leadership takeaways from these experiences.

Mentors provided commentary on their perspective of the coaches’ influence; they specifically emphasized the value of the coaching role on the overall internship experience. Naomi noted, “Obviously [the interns] meetings with [their coaches] had to go very well, because there were very few questions that they had to ask me.” Elizabeth highlighted the importance of accountability from the program, “you know Imani [the coach] is going to check in with you.” Rebecca described the coach as one of the most impactful components of the internship experience from her perspective as a mentor:

I think the program allows someone to have a person to support you along the way. To guide you through some of the things that you have to learn, helping you to think through some of the practices that you have to learn. And I think the impactful thing is just having someone there to coach, guide, and help you be more reflective in your practice. I think that’s the most impactful part.

As evidenced by the mentors’ reflections, the coach was instrumental in moving the triad towards achieving the overarching leadership development goals of the program throughout the internship.

The interns reflected on their experiences with the coaches and how those conversations shaped their internship year. Molly expressed appreciation for having regular communication with her coach by stating, “Dave is the best. When I say he kept me grounded. I could be transparent with Dave, regardless of race and age … We were together every week. We talked every week.” Likewise, Christopher shared:

He [the coach] would come to school, and he and I would use my classroom, and we would talk about, okay, these are the things that’s coming up that you need to make sure you have done. Do you need any help, any clarification on these things that are coming up that you need to complete? Or we would meet with Naomi, myself, and him. We would kind of sit down and talk about things. What’s working, anything you need clarity on, anything that’s not working, so typically as relates to the internship. Those are the types of conversations we would have but, like I said, in addition to those, we would have other conversations as well, that just kind of helped me stay grounded and stay sane.

Across the interns, there was consistent appreciation for how the coach helped guide the candidate through their internship. Although the program was stressful due to coursework, full-time employment, and taking on new leadership responsibilities, the coaches helped the interns remain steady and focused.

The coach’s role as a nonjudgemental guide was recognized by the interns. For example, Rhonda stressed the importance of having a trusted confidant outside of the building to troubleshoot specific internship experiences:

I would tell her my experiences with some teachers or some things that I felt like I did not want to address with my Mentor, because again, when the internship ended, I still have to work here. So there were some things that I got to bounce off of her that I didn’t necessarily maybe bounce off of them. So I think having that support was good, because of the questions that I could ask.

Likewise, the coach served as a trusted advisor who served as a liaison with the program itself. Rhonda explained how her coach provided a more direct connection to program leaders, “Sometimes I don’t want to ask a question [to program leaders] and seem stupid. So I would ask her [the coach], and then I guess she could ask during the group meeting.” Rhonda expressed gratitude for having a coach who could serve as a link between herself as the intern and program leaders.

Beyond the logistical components and the confidential support provided by the coaches, interns went on to highlight how coaches effectively facilitated their leadership growth through the coaching relationship. Tiffany shared that she loved her coach and that her coach “had me think about different things or see different things in different ways.” In addition to coaches helping the interns develop a leadership lens through challenging them to consider alternative perspectives, they also encouraged development through engagement in leadership activities. In some instances, the coach might direct the intern to participate in leadership activities that were outside their comfort zone, or lead the intern to a decision through reflective questioning. Tiffany described how her coach used the directive approach:

She made me step out of my comfort zone…I can talk in front of kids all day, but I hate talking in front of adults. And so, you know, she would be like, ‘Well, Tiffany, you need to present at the next staff meeting, or you need to do this’… making me speak in front of adults which worked out great…I laugh about it now, but I knew I needed to get out of that mode of not wanting to speak in front of the staff…I can tell now, when I’m uncomfortable I rise to the occasion…Imani, she always would just kind of push that envelope for me, and I appreciate that.

While Robert was supported by the same coach, he explained that the reflective questioning approach worked for him rather than being directed to do specific tasks. He shared that Imani held routine check-ins to go over his performance on the principal standards. Through these meetings, they would focus:

on different areas where maybe there’s zeros and ones. And how can we progress this? What’s your certain goal? I think we had the right goals for each professional standard. Here’s your goal that you wrote. How can we improve this goal to get you where we really need to be? Having those intentional conversations is moving me in the right direction.

This facilitative approach through questioning enabled Robert to make action steps to accomplish goals and move forward in his leadership development.

Numerous candidates reflected on how their post-degree coach helped them navigate the hiring process and establish goals in their new roles. Rhonda explained:

I think he has been really great because he’s kept me motivated, and without that check-in or check and balance I would keep putting things off. Just keep procrastinating, keep procrastinating. Having him kind of eliminates, or it puts a limit on my procrastination, you know I’m like, oh, I’m about to meet with him. Let me get this resume. Let me get this cover letter done … So he’s like, send it to me. Let me look over it, and then he provides feedback, so he has definitely kept me on track.

Robert also discussed doing interview preparation with his post-degree coach, which included talking through examples of potential responses to interview questions and getting feedback for alternative ways of demonstrating his leadership experiences while pursuing leadership positions.

Across all interview participants, the crucial role coaches play in both mentor and intern/alumni development was consistently reiterated. Coaches themselves recognized ways in which leadership coaching would have been beneficial in their leadership trajectory. For example, when reflecting on his own leadership journey, Paul stated, “I think a lot of times, I went into executive positions, including the superintendency, where coaching and feedback and bouncing things off someone else in a confidential manner have been helpful, extremely helpful for me.” Ultimately, the coach helped leaders navigate their careers by developing their leadership potential, and by serving as a trusted external support that offered a space for leaders to take risks and be vulnerable.

#### Program Implications Regarding the Coaching Role

Data from this one school leader preparation program indicate the importance of the coaching role. As leadership programs make decisions about including a coach to support the intern, they may consider how these individuals can be used to deepen the connection between the program itself and the PK-12 school setting. School leader preparation programs may consider not only the leadership background and expertise coaches come with, but also their skill in engaging in reflective practices and a willingness to advocate for the needs of those they support. Preparation programs should consider implementing coaching, as coaches provide an additional space for interns to develop as they apply theory to their leadership practice.

### 5.2. The Triad Relationship

The strength of the triad relationship varied across interview participants. The relationship coaches developed with the mentors impacted the triad’s ability to work together to further the intern’s development. Through a shared understanding of the coaching role, the intern, mentor, and coach had authentic conversations to help the intern work toward accomplishing their goals. While the program provided guidance regarding monthly triad meetings, there were no requirements for the coach and mentor to connect beyond these meetings; this led to differing experiences. As a coach, Dave emphasized the importance of conveying “that we’re kind of a cooperative triad, if you will, in terms of getting the job done.”

The mentors shared that they felt the cooperative triad was important to the development of the intern. Naomi emphasized the power of being in sync as a triad and the value of being able to bounce ideas off of each other. Beyond the triad, Naomi emphasized the value of interns being supported by program leaders and university professors. Naomi viewed this level of support as being instrumental in the development of the intern: “I think the influence is that when you have a supportive group and know that you can reach out to different people. That to me is a big impact.” Elizabeth reiterated this perspective by stating, “There’s all these layers of support and accountability to ensure that the experiences are had.” Furthermore, Cathy shared:

I think it’s awesome that [Tiffany] had not only the coach, but also you know me as her supervisor and mentor, kind of supporting her and guiding her, and that she could balance it between, like me, being her supervisor, but also her mentor, but also having someone on the outside being the coach that can kind of like see her, hear her point of view, and help her to strategize.

In their monthly meetings, the coach kept the triad on track and addressed the individual needs of the intern. For example, Rebecca shared, “That [it] was a great support having Dave there to guide us through some of our conversations, to keep us on task.” Another mentor explained how the coach:

would push me even further to think you know, and ask questions outside of the day to day, because it’s very easy when you’re in this role, to just tell people what to do and we didn’t want it to be, this is what you need to do. We wanted her [the intern] to establish her own leadership practices as well. So [the coach] helped tremendously with that.

Several factors influenced coaches’ relationships with the mentors such as proximity and trust; face-to-face meetings were influential for interns’ development. Carl emphasized geographic proximity as a way to develop a meaningful relationship with his intern’s mentor:

My proximity … just being right down the street from that duo, we worked on different things together because we serve a similar population. So I would see [the mentor] in other walks of the job. But if it wasn’t for that, I don’t know that I would have built a relationship with the mentors.

Coaches also noted the importance of explaining the structure and purpose of their role as a way to develop trust. For example, Lucy shared that in developing a relationship with the mentor, who was a new building principal, part of her role was to explain the program and that she was “there for the intern, but I’m not there to judge [the mentor].” She also shared that once the mentor “realized that I was willing to listen and not necessarily give advice…I think he realized that if he needed to, he could tap on me for a question or to bounce an idea off me.” The non-evaluative nature of the external coach created a trusting space and promoted a willingness by interns and mentors alike to ask for authentic feedback and help. The coach’s role also developed the building principal’s leadership practice, ultimately benefiting the school holistically.

Other coaches maintained a more surface level relationship with the mentors. For example, Matthew described his experience:

I felt like the relationship was good with [the mentors]. I didn’t feel like I developed as deep a relationship with those as I did with the interns…It was more collegial and professional and didn’t have the depth, the more facilitative role and the depth that you would have with the interns.

The differences in relationships could be due to a variety of factors, such as the level of experience of the mentor and the coach, or the demands of the school. Additionally, the dynamics could be influenced by whether or not the coach intentionally established a deeper relationship with the mentor.

Coaches providing post-graduation support found it more challenging to develop a relationship with building principals or supervisors. Paul shared, “I’ve not had a lot of contact with the lead principal.” However, he acknowledged that it was his responsibility to develop that relationship and reflected on how he would do that in the future: “I need to be a little more intentional about doing that. That’s not on the program that’s on me.” Luke also shared that developing a strong relationship with the building administrator was difficult. He shared that the relationship is “a matter of saying hello, having them know about the program, talking up the program.” He then noted that he wished program leaders “would train us to have an elevator speech about the program to pass on” to make this part of his role easier to accomplish. Navigating establishing a relationship with the supervisor of alumni requires a level of nuance since they are no longer interns but active leaders in official positions.

#### 5.2.1. Filling the Gaps

The cooperative triad model ensured that the intern received multiple layers of support to accomplish their goals. Coaches found that they were able to fill gaps in learning when the mentor was unable to provide the necessary experiences. Imani stated, “I think if they don’t have a strong mentor, then everything just falls to the wayside because the mentors are really creating the in-field experience for them.” She went on to explain the coach’s supportive role in these instances. Imani highlighted:

Even when their mentor wasn’t strong, you had this coach that was stepping in to support. I think just having those two layers, how many programs can say they have two layers of support where it’s not just, oh you have your in-field mentor, but you have your in-field mentor and then you have a coach from the program.

Lucy shared a similar experience. She explained, “Ultimately, [the mentor] wasn’t creating the time and opportunities that the intern really needed.” Lucy noted that even with a well-laid plan, the demands of school leadership can disrupt the internship. She shared:

I think there was always the intention to provide her [the intern] with the experiences. And then, once people got into the flow of the school year and got busy, some of the experiences that she needed. I don’t know if they actually happened, because oh, we just did it, or that meeting got cancelled or so. There were just like life, events that came up that kind of made it sometimes a challenge to get.

Imani also discussed the importance of making sure interns received the mentoring they need while being mindful of the mentor’s complex and time-consuming responsibilities leading a school:

It’s harder to push with a mentor than an intern. I found in my experience, because the mentors are building principals and they have so much on their plates, so sometimes I felt like I had to be more understanding of any gaps that may occur, or any things that may have fallen through the cracks because they are also running a whole building. That’s just something I struggled with personally in that role is, I want to hold them accountable and make sure they’re able to fulfill the role of a mentor, but at the same time I had to acknowledge that they’re building principals.

At times, the demands of school leadership led the mentors to rely too heavily on the interns to complete tasks beyond the requirements of the internship. Dave shared:

If a principal is expecting too much of the intern, which I can understand would be a temptation, especially if they don’t have an assistant principal or dean depending on the school system, it would be my job to say is there any way we can help someone so they [the intern] can focus more on the internship requirements?

Similar coaching conversations occurred when the interns were given too few responsibilities or when their tasks focused on only one component of leadership. Coaches advocated for interns and simultaneously provided a trustworthy relationship throughout their internship. For example, as an intern, Rhonda explained how the added layer of the coach in a triad relationship positively influenced her individual growth when processing challenging experiences. She stated:

Having meetings with my coach helped because I could talk about some things that I might not have felt comfortable talking to my principal about. I could talk to my coach about like I saw this, but I’m not quite sure if that’s right, and I could always bounce that back from my coaches, and when we would meet, sometimes we would talk about our day, or talk about difficult situations that we had. So that helped a lot.

Knowing that interns like Rhonda had an external support they could trust to discuss the more intricate details of real-life scenarios facilitated deeper reflections through honest discussion. Coaches not only provided opportunities for interns that their mentors could not, but also built trustworthy relationships that advocated for interns’ best interests.

#### 5.2.2. Impact on the Mentor

While the coach’s role is not to provide direct support to the mentor through the triad model, several mentors discussed the benefits of the coach on the mentors themselves. For example, Cathy shared that the coach helped her become a better mentor, specifically stating, “[the coach] may see what needs to be done to support [the intern], but [the coach] said it in a way that was not offensive or accusatory, but more just holistic and appropriate and comfortable for me.” When describing the overall relational dynamics, Rebecca explained, “My relationship with Dave was wonderful…I really enjoyed the feedback he gave to us as a team, and then I was very appreciative of some of the things that he could share from his experience with me as a leader.” She went on to say, “So his relationship was more like he was a mentor to me, and also, helping me to get through the year of being reflective of my practice. So I appreciate that a lot.”

In addition to improving their mentoring practice, several mentors noted that the coach helped them become better, more reflective leaders. Rebecca, a veteran principal, explained that the coach added value as an outside perspective to her work. She shared that the coach:

really pushed my thinking. He actually, in some of the conversations that we had, allowed me to go back and question myself…Having someone when you’re in the work, sometimes things look different for you when it’s somebody from the outside.

Naomi, another veteran principal and mentor, shared that the program’s coaches helped her grow in her role. She stated, “Dave and Carl were amazing as well. Them meeting with me pushed me to kind of think of things in a different way. Likewise, Natasha expressed, “I had what I felt like was my own mentor throughout the process.” Although the coach’s primary objective was to support the intern, mentor anecdotes clearly explain how coaching also positively influenced their leadership skills.

#### 5.2.3. Program Implications Regarding the Triad

School leader preparation programs can use the triad model in order to coach and support both the intern and mentor. When designing their model, programs should prioritize the coaches’ geographic proximity, face-to-face contact, and meeting structures. If programs are unable to employ a coach as part of a cooperative triad, programs will need to consider how they will facilitate a well-rounded experience for the intern in the event the mentor is not available or skilled to provide the right leadership development opportunities.

### 5.3. Typical Coaching Session

Coaching sessions during the internship year were guided by a series of open-ended questions addressing interns’ leadership development and performance toward their field-based projects, their experiences in specific areas of leadership, and work toward their goals. In addition to the program’s standard coaching session questions, the gradual release calendar, an individualized program dashboard with embedded internship requirements, was used to frame the monthly coaching sessions during which the coach, mentor, and intern met as a cooperative triad to discuss the intern’s progress in the program. Overall, the coaches provided a consistent overview of the typical flow of a coaching session. For example, Matthew shared:

The typical coaching session had an agenda. I would look at the questions from the gradual release and try to go over that, and then the notes that I would have from our monthly leadership meetings…I always want to hear some of the creative gifts they were bringing to the table. Tell me some of the cool things that you’re doing.

In some instances, the coach excluded the mentor from meetings to hear from the intern. Allison shared, “He [the coach] would meet with me individually before he met with me and my mentor.” She went on to explain that sometimes, the coach held meetings with just the mentor. Similarly, Rhonda’s coach held more frequent meetings. While the mentor attended the required monthly meeting, the additional meetings provided time for Rhonda and her coach to meet individually. Shifting between meeting as a triad and meeting one-on-one with the intern or the mentor allowed the coach to gain a better understanding of the intern’s needs to determine how they could best be supported.

Preparation for triad meetings or one-on-one meetings was accomplished by reviewing the intern’s progress on the student dashboard. By reviewing the intern’s dashboard, the coaches were ready to discuss action items and next steps during the meeting. Carl described this process as follows:

I prepare by looking at anything that’s missing or anything that is incomplete. And I make sure that I hit on those, and so we go tab by tab on the bottom of the gradual release form. So we just verify that within each tab, you’re as up to date as you should be. If there’s something that we need to discuss, something that we just need to brainstorm on, we have an opportunity to do that. And I ask the mentor if there’s something they would like to see from the mentee.

Dave explained that he reviewed the intern’s progress in their gradual release calendar to conduct a pre-coaching session conversation:

On the Monday before our Thursday meeting, I will find out what time I can call the intern and take care of some housekeeping things, talk about deadlines and that sort of thing. Nothing really heavy duty, so that when we meet, I am able to stick to the agenda that I had actually sent them by email.

The structured agenda coupled with the live access to the gradual release calendar provided coaches with a consistent way to ensure the triad meetings focused on supporting the intern’s progress towards their individualized goals and addressing barriers to accessing required experiences.

After graduating from the program, candidates continued to receive coaching support for two years. Coaches supporting alumni were asked to meet face-to-face once per month and hold online coaching sessions between visits. At times alumni schedules were challenging to work around, and in some instances, in-person coaching sessions were not possible. However, visiting the alumni on the job was helpful for the coaches. Luke shared:

I need to go [in-person] at least every other time…I don’t want to be a surprise or a thorn in the side. It’s during their planning or after school, not in the way. And it’s never punitive that I’m coming to visit.

He went on to explain that seeing the graduates he supported in their workplace helped him get a better sense of how they were performing as new leaders, and allowed him to modify his coaching sessions to address the graduates’ immediate needs. Similarly, mentors and interns noted the importance of in-person coaching during the internship year. As a mentor, Naomi believed that face-to-face coaching sessions were a valuable component of the internship; she stated, “I think it’s powerful when you come into a building.” In-person visits were so fundamental to Naomi that she further explained her willingness to adjust her busy schedule as a principal accordingly. Molly reflected on her time as an intern and expressed appreciation for her coach and, “the in-person visits to see … your actual working environment and what you’re doing.” She further shared that as a graduate, “we’re missing that in-person, coming in, and that support” and expressed a desire for “some more hands-on support after graduation.” Molly explained:

Because of the in-person coaching, the [coach] could see something…even though I’m sitting here and I’m saying I need help with X, Y, and Z, there might be something that, ‘Oh, Molly, you could have handled this better if you would have done ABCD.’

Therefore, in an effort to support alumni in their new roles, seeing them in action through face-to-face coaching may help the coach better understand the graduate’s needs and inform the focus of the coaching sessions.

As alumni acquired leadership positions, the coaching and skill development remained focused on their continual development while being responsive to their unique job responsibilities and career aspirations. Paul, a coach, explained:

We normally start out, how’s your week going? And we segue back to any issues or anything that we had talked about previously, any concerns or projects or processes that they were working on. We take the last session and segue into this session. Then we talk about the new goals and where they are with their goals, and then we talk about is there anything new.

As a graduate of the program, Robert initially received a lot of direct support from his coach through interview preparation and troubleshooting specific scenarios within the work. Now that he is in his leadership role, Robert explained that he and his coach “kind of meet on an as-needed basis. We check in about various things. He’ll text me and just make sure I’m doing ok.” After catching up on their lives, the coach shifts the focus of the conversation back to Robert and how he is doing in his leadership role. Robert shared that he appreciates the informal nature of their coaching relationship and that he continues to feel supported. Although coaching sessions were often structured, interviews surfaced that coaches adapted their sessions to respond to the preferences of interns and alumni alike.

### 5.4. The Structure and Fluidity of Coaching

Throughout our interviews across the triad of individuals there was an emphasis among participants to maintain a balance between creating a clear structure and meeting the real-time needs of the individual student. To do so requires the coaches to be flexible when needed and work with the program team to make adjustments, including adding supports based on the unique needs of the triad. Paul noted how he established a structure from the beginning by stating:

This is what we’re going to talk about, this is not what we’re going to talk about. But at the same time, you keep your conversations freeform…you allow them and you to open the floor to discuss things. Also, with this process there are defined goals.

He went on to highlight the ways in which coaches need to be flexible in their role:

You do whatever you need to do to pivot with whatever they want to discuss and talk about. So a free-flowing conversation was emphasized. But at the same time, you also try to keep it structured to where you’re meeting their needs.

Matthew highlighted the structure provided by the gradual release calendar by stating, “It is a masterpiece…I’ve never seen anything so efficient in my whole life, but there’s a lot to it.” Matthew discussed how he used the gradual release calendar as a guide for their coaching sessions, but first “would get the conversation started before we start talking about hours and meeting the goals and all those kinds of things” by asking about positive things happening in the interns’ work and personal lives. The gradual release calendar helped Matthew have an understanding of areas where the interns were having trouble accessing experiences such as facilities management, budget planning, or master scheduling; this allowed him to go into more detail about his leadership experiences in these areas while also working on a detailed plan for the intern to gain necessary experiences.

#### 5.4.1. Meeting Individualized Needs

Although the internship requirements were the same for everyone, different individuals required different levels of support from their coaches. Intern needs were often driven by the strength of their mentor and the relationship between the two. For example, Dave shared that an intern he supported “needed very little support from me because she has a very strong work relationship with her principal…So it’s always driven by need.” Imani noted that one of her interns across the two years she served as a coach needed additional support due to limited availability of the mentor. As such, Imani increased the number of times she met with the intern to ensure the intern was engaged in a breadth of leadership experiences. Additionally, Imani went above and beyond by modeling certain aspects of the required activities. She explained:

I think what is most beneficial is that [the interns] are really getting the coaching and support on leadership activities through their mentor. But in my case, I don’t think that my mentors were either strong enough or had time to do that, and so I was stepping in more frequently than you would expect a coach to be stepping in.

She also shared that she considered not only areas in which they needed support in the moment, but also their plans for the future. Imani stated, “I also tried to think about, where does this person want to be? So thinking about that lens and their leadership experience…meeting their individual needs of what they eventually want to become.”

In another situation, a school leader mentor left mid-year; program staff worked with the school district’s central office to provide mentorship support from a principal director who was instrumental in the intern’s career development. Due to this unique situation, the coach responded to the support the intern needed by increasing the frequency of meetings. Delores, the intern, further explained:

I requested that I meet with him [the coach] twice a month instead of once a month. I told him I needed more support. We would have informal sessions where we would just talk about whatever we needed to talk about, and then the next session would be more formal.

As interns matriculated through their internship, the program staff had to stay attuned and make adjustments to provide the right support that met the interns’ unique needs.

The idea of meeting aspiring leaders where they are continued as a theme in the coaching of alumni as Paul described:

I have three very distinct personalities I’m working with right now, and some are more confident than others. Some are at different places in their lives. Some are reluctant leaders…A lot of times you have to convince them that they can do it and then provide them with the tools that they feel like they need going into a job interview to be successful. Some are naturally inclined to be competent and charge for it, and others have to be kind of nudged and pushed, and persuaded to do it, so you handle that depending on the individual.

Luke explained the importance of understanding the relationship between the alumni and their new supervisor, such as the school principal. This helped him determine how much individualized support his graduates may need as they progress forward in their careers. He shared:

The introduction of the [supervisor] is important…I thought, ‘I need to see you in your environment in order to help you…I really always want to understand the relationship they have with their school principal. Is this principal wanting to help their career? Are they growing them, or are they holding them?

Throughout the program and even following graduation, coaching needed to be responsive to each individual served. By tailoring coaching support based on school context and leadership readiness, interns and alumni experienced unique leadership development.

#### 5.4.2. Program Implications Regarding Coaching Structures

When implementing coaching as a lever for leadership development, programs ought to balance designing structured systems of support with real-time adjustments based on the intern or graduate’s needs and preferences. For example, programs can ask coaches to facilitate meetings that consistently review program milestones, set goals, and monitor progress. Likewise, programs should encourage coaches to problem-solve with one another and adjust the coaching support provided accordingly.

### 5.5. Coach Preparation & Program Growth

Several of the coaches provided recommendations for future selection and training of coaches in the program. Prior experience as a building administrator was a key recommendation for selection. Imani stated, “It’s about picking the right coaches, those really experienced administrators that have been building principals…Their expertise that they bring is really the most important thing.” She went on to explain that this experience is important because “they’re not really just coaching an intern and making the best next generation leader, but they’re coaching that principal too.” Having a coach who has served in the role of building principal makes it easier for them to create a bond with the mentor. Naomi described that as a mentor, she appreciated the conversations she had with the coaches who were retired principals. She shared, “They were easy for me to talk to. I said, ‘Hey, let me tell you what happened to me today,’ and they’re like, ‘I remember those days as a leader.’” She went on to elaborate that while she was able to have conversations about work-related issues with her assistant principals, discussing the same topics with the coaches was often a more meaningful experience. Interns too described the significance of coaches having prior leadership experience. Tiffany stated that because of her coach’s prior building-level leadership position, “it’s very easy to talk to him” about things she was encountering in her new leadership role.

In addition to being experienced leaders, several other factors were recognized as being important in selecting and retaining coaches for the program. Paul stated, “It does help to retain the coaches from year to year;” as coaches successively practiced their role they would refine their coaching skills and have a greater impact on those they support. Geographical location was another consideration, as in-person coaching sessions were beneficial. Lucy explained, “I’m supporting two schools, but they’re in the district that I live in, so it’s going to be much easier for me to do on-site support. … I found being on-site very valuable.” Beyond proximity to the interns and graduates they supported, the assigned school level was another factor to consider when selecting coaches. Dave, a former elementary school principal, shared, “I was coaching one of the interns at the high school level, and high school is foreign territory to me…I wasn’t sure exactly how to approach that because that’s a whole different ball game.” Interns also shared having similar backgrounds and work experiences as their coaches. Robert stated that his coach “has done work very similar to me in special education, leadership. So being aligned with a leader that had similar experiences with me, could talk my language and understand my jargon, was very helpful.” Similarly, Christopher noted, “I love the real conversations I was able to have with Carl from multiple perspectives. Not only as it relates directly to the program. But also, as an African American male in education.” Beyond selecting the right coaches for the role, pairing coaches and interns with shared backgrounds helped establish trust and facilitate conversation. Delores reiterated this point by explaining that she and her coach:

Have a great relationship … I think he’s a good match for me. But you know, mentors and mentees they really need to be a good match. I think he’s a great match. He just gets it. I feel like he understands, with me starting a new position, what that may feel like … He’s very understanding. All the things that you would want in a mentor or a coach.

While coach selection and pairing are critical when establishing the coaching triad, the training coaches receive influences their preparedness when supporting the next generation of leaders.

#### 5.5.1. Practicing the Role

As the program continued to evolve, multiple coaches expressed a desire to practice their coaching skills before diving into the role. Dave shared the importance of preparing coaches by stating:

When we get new coaches on board, none of us, including myself, should assume anything, no matter how good the orientation or training is for them, no matter how many of the seminars they go to. That’s still going to be different than actually implementing the gradual release calendar, actually in the buildings, in-person with the interns and mentors.

While coaches came with a vast amount of knowledge and experience as leaders, they wanted to ensure their coaching style and actions aligned with the desires and expectations of the program. For example, Carl shared, “I wasn’t sure how to navigate it [leading a triad meeting]… if there’s a chance to practice that before just jumping into it, that would be great.”

Matthew also indicated that he would have appreciated the opportunity to role play some of the required meetings and trouble shoot any of the common issues that arise throughout the internship year. Due to the complexities of the gradual release calendar, he stated:

Getting to know [the gradual release calendar] was what I was initially worried about more than anything else…If I could go through it again and learn how to use the gradual release, it would have been some scenario-based, and then writing it out, all those kinds of things where I did it the way I would have, where I would know exactly what it was. You’d maybe simulate that mentor-intern meeting, and then go back and write out the comments, and so forth…The more interactive you make it, the better off it would be.

Similarly, Lucy explained how she would have benefited from more concrete examples by stating:

If there were more exemplars of what they’re looking for specifically; you’re giving feedback, being able to view each other or have some type of exemplar, just so that you know that you are on the right track with the type of feedback and support that you’re giving.

As evidenced by the various comments provided, even with years of experience under their belt as leaders, the coaches wanted clear opportunities to practice and receive feedback on the core expectations of the program before diving into the coaching role.

#### 5.5.2. Coaching the Coaches

As coaches settled into their role, they relied on one another for guidance and expressed an interest for professional development beyond the initial orientation and training. Especially for new coaches, peer-to-peer development was welcomed and appreciated. For example, Carl shared how Dave, an experienced coach, supported him in the role. He stated, “I know he’s only done it like a year or so longer than me. Dave is on point and so if I ever have questions, I can reach out to him, and he is like super, super prepared.” Likewise, Matthew benefitted from Dave’s experience. He shared, “He was really helpful to me because he was available to me, and we kind of latched on each other…He helped me technically more than anything else…Make sure you do this. Make sure you do that.”

Beyond supporting each other as colleagues, the coaches reached out to program leaders requesting further development in the role. Paul shared:

We [coaches] were concerned about whether or not we were being effective. They [the program] brought in an executive coach…to kind of guide us for a few sessions and work with us just to make sure we were on track. That served two purposes. It validated some of the things we were already doing, but it also gave us some ideas for future coaching sessions that we could use…I felt prepared going in, but you know at a certain point, you kind of look inward. And I can be better at this, and we see [the program] kind of went the extra mile to provide us with that.

Paul emphasized the value in coaches developing each other’s coaching skills, as well as program leaders listening and being responsive to the needs and desires of the coaches. Luke echoed appreciation for the team approach and added coaching development sessions by stating:

I really appreciate the monthly meetings because for example, last year we talked about the concept of being a better mentor and what this should be. The [program] brought in a specialist for us and we had a session just for us, and that professional development was great.

Beyond peer and program support, Matthew made a salient point about coaching development: “It’s like anything else, the first year you do it, it almost takes you a cycle to get used to something.” Coaches honed their skills not only through one another and the tailored professional development program leaders sought to provide, but also through continuous and repeated practice.

#### 5.5.3. Program Implications Regarding Coach Preparation

Rigorous criteria can be used in the selection of coaches; however, demonstrating excellent leadership competences in an interview or application does not always translate to proficient coaching. Therefore, just as there are best practices for developing educators, school leader programs should identify and implement best practices for developing coaches such as repeated practice, role-play, providing examples, executive coaching sessions, and peer collaboration. In response to coaches’ needs, programs should provide training that deepens their coaching strategies.

### 5.6. Coaching Beyond Graduation

As previously explained, alumni receive post-degree coaching as part of the program for two years following graduation. We heard from coaches and alumni about the clear value add of this extended support, ranging from practical guidance to accountability for career goals, and access to a professional network. Paul, a coach, noted how post-degree coaching allowed for focused time on preparing graduates for job interviews:

There’s an opportunity for them to participate in mock interviews, on-site interviews…You walk into an interview, and you basically don’t know what to expect. You don’t know what questions they’re going to ask, and you don’t have those materialized or formatted in your mind. This gives them an opportunity to practice. Shake the nerves off and excel.

Depending on the candidate, this example highlights how post-degree coaching further ensures that alumni are pushing themselves and prepared for the next step in their career.

Coaches and alumni acknowledged that their relationship extended beyond the life of the set two-year commitment. For example, Lucy explained her sustained relationship with a former intern:

She’s still in touch with me now. She came and spent a day with me in June and reached out to let me know that she had an interview coming up. We’re still in contact even though I’m not required to be in contact.

As a new leader, continuing to learn from an experienced coach, specifically through on-site visits at an excelling school, is something that may not have occurred without an already established coaching relationship.

Likewise, program graduates noted that their coaches remained committed to serving as a professional resource and sounding board as they began their leadership careers. Allison shared that her coach encouraged her to keep growing in her career and be open to new opportunities. She stated, “He has encouraged me to keep all my stuff updated, my cover letter and resume, in the event that I want to do something else.” She went on to explain that her coach is her go-to person when seeking advice about potentially going back to school to obtain a doctorate: “He’s been a good resource with questions that I might have in that process as well.” Similarly, Tiffany and Robert both reached out to their coach to share what was going on in their personal and professional lives. Tiffany described how her coach encouraged her to connect with fellow program graduates because of the loneliness that leaders can feel. Rhonda also stated that her coach continues to support and advise her and even visits her at her school: “Her [coach] being a former building administrator helped. She would be like, ‘you can’t do that’, or ‘you need to take care of yourself when you leave here.’” Coaches and alumni alike described the importance of providing coaching beyond the internship year. As new leaders, alumni often navigate new experiences and face challenges alone. Post-degree coaching appeared to be just as important as coaching during the internship year because it provided alumni with a thought partner, actionable insights, and comradery.

#### Program Implications Regarding Post Degree Coaching

Following the internship experience, aspiring school leaders are stepping into or seeking a new administrative role where coaching continues to be an important component. As leadership programs design a multi-year support plan, they will want to consider how to scaffold coaching support to meet the evolving trajectory of their graduates. For example, programs may want to consider how coaches can maintain high visibility and impact, while also streamlining systems and structures that feel relevant to current leadership demands.

## 6. Discussion

Through semi-structured interviews with 19 Eastern School Leader Preparation program participants, including seven coaches, seven interns/alumni, and five mentors, this study explored how the coaching role is conceptualized, and the factors that shape the leadership program’s implementation and stakeholder development. Researchers aimed to address questions related to the coaching position, structures, practices, and influence. Study findings align with existing research and draw additional conclusions about coach selection and pairing, coaching structures that benefit leadership development, and the influence of coaching on program participants. This study also gathered information about what supports coaches’ development, including what resources or training may be necessary to strengthen the coach’s comprehension of their role and efficacy.

### 6.1. Coach Selection & Pairing

Existing literature provides differing viewpoints as to whether coaches should be internal (Wise & Cavazos, 2017) or external (Fusarelli & Fusarelli, 2023; Kappler-Hewitt et al., 2020) to the school district they support. Others suggest that skillset is more important than whether a coach is internal or external (Salavert, 2015). Our findings support external coaches in leadership development. Establishing trust through a confidential relationship with an outside coach allowed the interns to have transparent conversations they were not always comfortable having with their mentors (Fusarelli & Fusarelli, 2023; Stevenson, 2017; van Nieuwerburgh et al., 2020; Wise & Hammack, 2011). While not a major theme of the findings, the power of identity connections, such as race or background experiences, fostered safety between the intern and coach (Lochmiller, 2024). Similarly, because coaches held leadership roles outside of the interns’ school districts, which limited concerns related to employment evaluations, the interns could openly discuss a wide range of topics, including professional challenges and even issues from their personal lives (Fusarelli & Fusarelli, 2023; Taylor, 2008).

When determining coach pairings, program leaders should not only consider coaches’ external roles and identity markers, but also the leadership experiences they bring with them (Lochmiller, 2024; Master et al., 2022). As Dave shared, his experience was at the elementary level, so that is where he felt the most comfortable; pairing him with a high school-level intern led to a greater learning curve versus at the elementary school where he could more efficiently execute his role. Another consideration highlighted in the findings and supported by the literature is the coaches’ proximity to the school sites, ensuring face-to-face coaching sessions on a consistent, ongoing basis (Kappler-Hewitt et al., 2020). Both interns and coaches emphasized the value of being in the school building together to foster a trusting, productive relationship. In-person contact enabled coaches to understand the intern’s school context, observe their engagement with the school community, and see their leadership development in action (Cosner et al., 2018).

By collaborating with district representatives, program staff not only learned about the school’s context, but also the building leaders who would serve as mentors. That knowledge helped them determine the right coach to support the mentor-intern pair based on their anticipated needs. When mentors were unable to provide the leadership experiences the interns needed due to job constraints or gaps in knowledge or skill, coaches were ready to facilitate valuable leadership opportunities (Cosner & De Voto, 2023; Cosner et al., 2018; Drake et al., 2023). While ideally university-school district partnerships work together to determine the school and mentor placement of each intern, at times the school district leaders determine which building principals will serve as mentors, and as such the program is unable to have a say in which intern-mentor pairings will work best. However, program leaders unilaterally select coaches and determine which mentor-intern pair they will support through a cooperative triad. Keeping in mind the coaches’ backgrounds, experiences, and proximity helps program leaders create successful cooperative triads that meet the needs of the intern, mentor, and school community (Lochmiller, 2024; Kappler-Hewitt et al., 2020).

### 6.2. Coaching Structures

Findings highlight the importance of regular, in-person meetings that are structured yet flexible (Kappler-Hewitt et al., 2020). Monthly meetings facilitated through an established agenda helped the triad keep the intern on track by examining their progress in completing required tasks outlined in their gradual release calendar. Coaches in a triad model have a birds-eye view of the mentor-intern pair, allowing them to clearly see challenges and address them through an adaptive approach (Heifetz & Laurie, 1997). The triad structure created an environment of accountability for both the mentor and the intern which ensured that regular progress was made towards individualized goals as well as the overarching goals of the internship (Cosner & De Voto, 2023; Cosner et al., 2018; Snodgrass Rangel et al., 2024). The meetings also aligned the mentor’s and coach’s approach and allowed them to have a shared understanding of the intern’s progress. Likewise, the meetings allowed mentors and coaches to know when they should reach out to their professional networks to best support the intern with resources to achieve their goals (Cosner et al., 2018). While the structured meetings addressed topics required by the program, flexibility was encouraged to respond to the needs of the intern and engage in discussions centered on spontaneous events occurring in the school environment. Utilizing virtual platforms, such as a digital portfolio or reflections, provides the coach with current internship data necessary to support ongoing leadership development (Drake et al., 2023). Aligned with the foundational work of Cosner and De Voto (2023), our data revealed the important role the coach plays in ensuring the intern completes tasks and meets their goals through reflective conversations within a triad model.

Coaches working with alumni were also encouraged to hold regular meetings to help them transition into leadership roles. Unlike the cooperative triad developed during the internship year, the program was cautious about setting a firm commitment with the alumni’s new supervisor as a way to honor their leadership role. Yet, coaches still expressed a desire to establish some connection with the supervisor in order to understand how to effectively coach their graduates in their current position and towards future opportunities. While being mindful of the existing employee-to-supervisor relationship, establishing even an initial meeting to explain the coach’s purpose and presence in the building could yield a more fruitful post-degree experience. Similar to coaching interns, alumni required flexibility in the coaching structure and communication. Unplanned communication was valued by alumni because, as practicing leaders, they experienced questions and challenges at times that required immediate attention. In alignment with previous research, interns and alumni alike benefit from intermittent communication through various means, such as phone calls and text messages (Cosner et al., 2018; Kappler-Hewitt et al., 2020). Programs may consider encouraging coaches to engage in this type of impromptu communication to cultivate a trusting relationship between members of the triad (Snodgrass Rangel et al., 2024).

### 6.3. The Influence of Coaching

The ultimate goal of leadership coaching is to develop the intern to become a skilled and confident school leader. While research indicates that building principals can help develop school leader interns through mentorship (Thessin et al., 2020), our findings revealed that mentors are not always able to provide them with the leadership opportunities needed. The addition of an external coach through the triad model can fill these gaps, as coaches are able to use their professional experience and network to facilitate leadership opportunities for the intern. Grounded in selecting the right coach for the mentor-intern pair and the effective structures employed by the program, interns shared anecdotes of how coaches targeted their areas for development through active listening, reflection, and questioning (Patrick et al., 2021). These purposeful coaching strategies align to the work of Augustine-Shaw and Reilly (2017) and Stevenson (2017), and ultimately equip interns to become competent leaders.

Mentor principals also benefited from participation in the cooperative triad (Snodgrass Rangel et al., 2024). Mentors consistently expressed appreciation for how coaches helped them improve both their mentoring skills and leadership practice. The varied leadership experiences coaches brought to the role helped mentors see things from a new perspective. Because of the strong, positive relationships developed across cooperative triads, building principals often saw the coach as their own mentor and felt that the coaches pushed them to grow in their leadership roles. Previous research highlights the rationale for providing external leadership coaching to practicing principals (Darling-Hammond et al., 2022). As such, district partners might consider utilizing external coaching to further develop leaders as a way to positively impact the school community (Boon, 2022; Su-Keene & DeMatthews, 2022).

Existing research focuses on the added value of having school leader interns supported by either a mentor (Thessin et al., 2020) or a coach (Collins et al., 2025; Kappler-Hewitt et al., 2020; Wise & Cavazos, 2017) throughout the internship experience (Snodgrass Rangel et al., 2024). The findings presented throughout this article suggest that while having either a mentor or a coach is beneficial, the benefit from this study comes from the triad model (Cosner et al., 2018; Cosner & De Voto, 2023). Therefore, we extend this growing body of research by highlighting the value of engaging in a triad; having a dual support system for the intern also inadvertently supports the mentor. Within the context of this program, the triad approach created a unique development experience in which the intern observed and reflected on a model of leadership through their mentor, and gained tailored opportunities for coaching through their external coach. Establishing structures in which the triad can work together through meetings facilitated by the coach may help enhance the purpose, clarity, and consistency of the internship experience.

Similarly, post-graduation, alumni shared how program coaches supported them in their current leadership roles. Coaching shifted from a structured experience during the internship year to a more individualized experience responsive to the graduate’s priorities and real-time needs. Coaches not only supported alumni in their present positions but also helped them envision future leadership roles and held them accountable for setting goals and taking steps to achieve their aspirations. As they moved into new leadership positions, coaches encouraged alumni to build a professional network to increase their social capital (Uen et al., 2018) and avoid the isolation that is commonplace in leadership. Rogers and VanGronigen (2023) emphasize the importance of continuing the induction process beyond the preparation programs to further support alumni in their role socialization as new leaders. Continuing to support alumni immediately after their internship experience through coaching can help them move into leadership roles. As Bastian et al. (2025) found, individuals become less likely to become school leaders as time passes following graduation.

### 6.4. Support for Coaches & Continual Improvement

As Collins et al. (2025) highlight, there is a lack of research around structures for strengthening coaches’ practice. Our study adds to the field by examining how ESLP coaches were supported and developed, while also identifying how school preparation program leaders can and should work to continually develop in this area. While coaches come with a wealth of knowledge and expertise, they also require structured support from program staff to successfully perform their role. In the triad model, coaches provide support and guidance to interns and their mentors, while simultaneously engaging in conversations meant to challenge and further develop the pair. Although the program offered an onboarding orientation and monthly check-ins to support coaches, they requested additional training. Findings suggest that practicing how to use the gradual release calendar through role playing and other activities could boost coaches’ confidence when facilitating monthly cooperative triad meetings. Ongoing professional development opportunities for coaches could refine their coaching practice and promote common understanding and implementation as they work through problems of practice (Collins et al., 2025; Huggins et al., 2021). As a way to build the coaches’ skills and create a sense of community, Cosner and De Voto (2023) suggest opportunities such as engaging in a book study around a coaching concept.

Through regular meetings between the coaches and program leaders, coaches were able to share with one another how they have fostered relationships with participants and how they are adapting the program’s requirements to meet individual needs. The meetings also offered a natural place to troubleshoot concerns (Crow & Whiteman, 2016), such as limited mentor availability and intern challenges with completing internship requirements. For this reason, holding monthly check-ins with coaches creates structured opportunities to clarify expectations and collaboratively solve problems (Huggins et al., 2021). Continual improvement discussions created a space for the program team to listen to coaches’ feedback and make adjustments to improve quality and outcomes. Moving forward, programs should intentionally incorporate input from all stakeholders, including mentors, interns, and alumni, into their regular meetings in order to embrace continual improvement and maintain a responsiveness to the evolving needs of the field.

### 6.5. Limitations

Our research is limited by the young nature of the program, which, consequently, leads to a smaller number of participants and a limited longitudinal examination into the influence of coaching. Additionally, all coaches were external to the school districts they served. Considering the sample size of coaches, we strengthened our data analysis by triangulating findings across three groups of program stakeholders: coaches, mentors, and interns/alumni. The various perspectives of all stakeholders in turn offsets the program’s small pool of coaches. We address any further limitations regarding the influence of our personal biases on this study in our reflexivity statements within the methodology section above.

## 7. Conclusions

This study examined the conceptual understanding of the role of the coach across program participants, the structures and supports that contribute to the coach’s success, and the coach’s overall influence in the Eastern School Leader Preparation Program. Overall, findings confirmed that coaches not only provide a relational, trustworthy partnership with the intern and their mentor, but also push the intern’s leadership competencies to unlock potential which may have further consequences for the staff, students, and community they serve (Collins et al., 2025; Master et al., 2022). Notably, internship coaches maintain a non-evaluative, neutral position which invites the intern to be vulnerable, take risks, and ultimately develop their best leadership selves (Fusarelli & Fusarelli, 2023; Taylor, 2008). This study focused on one young school leadership program; while there was agreement across stakeholders regarding the benefit of coaches, as the program grows and more voices are heard, contradictory viewpoints may begin to emerge. Although limited in scope, the findings from this study may contribute to future research and program implementation.

As coaching continues to be applied in leadership development, school leader preparation programs and the research community could benefit from examining ways in which participating in coaching during an internship can inform the development of coaching skills within new and practicing leaders (Collins et al., 2025; Thomas et al., 2024). Likewise, research should explore how intentionally practicing learned coaching skills as an aspiring leader within their internship transfers to implementation as school leaders (Almager et al., 2021; Lewis & Jones, 2019). Because principals often need to utilize coaching components to develop and enhance the skills of teachers, maximizing their comfort level of engaging in coaching throughout their internship and early career experience may positively influence their ability to incorporate coaching practices into their leadership role. Due to limited existing research and limited findings from this study, another topic requiring further investigation is how to best support coaches’ development (Collins et al., 2025). For example, researchers can explore tactics and strategies that develop coaches’ skillsets in order to confidently and effectively execute their roles.

Additionally, researchers found a link between social capital theory and the findings of this study, where participants benefitted from a network of professionals to reflect with, troubleshoot, and learn from as they improve their practice (Cosner et al., 2018). Furthermore, human connection from a community of colleagues helps coaches and leaders’ morale, satisfaction, and longevity in the field (Rogers & VanGronigen, 2023). Preparation programs might consider ways in which they can embed coaching into school leadership development (Collins et al., 2025; Kappler-Hewitt et al., 2020) and collaborate with district partners to continue to provide coaching to program graduates (Fusarelli & Fusarelli, 2023; Su-Keene & DeMatthews, 2022). In this study, coaches played a role in the development of interns, alumni, and mentors alike. By integrating an external coach into a triad model, the coach supports both the intern and the mentor allowing the mentor to reflect on and evolve their leadership style while positively developing the leadership trajectory of the intern (Snodgrass Rangel et al., 2024). Unlike the often used coach-coachee or mentor-intern model which specifically develops the aspiring leader, the triad model demonstrates the potential for increased impact. As the coach guides the cooperative triad and helps develop both the aspiring and practicing leaders, the enhanced leadership presence may positively benefit the school community.

## Figures and Tables

**Table 1:** Research Question and Data Alignment.

Findings	Data Themes	Aligned Research Questions
5.1 The Role and Influence of the Coach	Coaches: Role definition/purpose; Mentors: Direct support to the intern Interns: The right amount of support; Learning from the coach; the coach pushing the intern	RQ1, RQ2, RQ3, RQ4
5.2 The Triad Relationship	Coaches: Relationship with building administrator; Triad Mentors: Triad Interns: Triad	RQ2, RQ3
5.2.1. Filling the Gap	Coaches: Relationship with building administrator; Triad; Mentor not providing opportunities/not being strong Mentors: Triad Interns: Balances with the mentor; Triad	RQ2, RQ3
5.2.2. Impact on the Mentor	Coaches: Relationship with building administrator; Mentors: Impact on the mentor	RQ3
5.3. Typical Coaching Session	Coaches: Typical coaching session Interns: Coaching meetings	RQ2
5.4. The Structure and Fluidity of Coaching	Coaches: Resident drives the conversation; Scheduling; Meeting them where they are; Flexibility & the triad Mentors: Flexibility Interns: Coaching meeting structure; Flexibility in meeting	RQ1, RQ2, RQ3, RQ4
5.4.1. Meeting Individualized Needs	Coaches: Resident drives the conversation; Scheduling; Meeting them where they are; Mentors: Flexibility Interns: Flexibility in meeting	RQ1, RQ2, RQ3, RQ4
5.5. Coach Preparation & Program Growth	Coaches: Support from the program; Recommendations; Additional training; Coach growth through training; Coach selection Mentors: Coaches were experienced Interns: Similarity in experience/demographics	RQ2
5.5.1. Practicing the Role	Coaches: Recommendations; Additional training; Coach growth through training	RQ2
5.5.2. Coaching the Coaches	Coaches: Recommendations; Additional training; Coach growth through training	RQ2
5.6. Coaching Beyond Graduation	Coaches: Continuing opportunities & relationship post-degree Mentors: Continued support after graduation Interns: Continued support; Resources & connections	RQ4

## Data Availability

The data presented in this article are not readily available due to the small sample size and potentially identifiable nature of the data. Requests to access the protocols should be directed to the corresponding author.

## References

[B1-behavsci-16-00798] Aguilar E. (2017). What’s the difference between coaching and mentoring? *Education Week*.

[B2-behavsci-16-00798] Aikens M. L., Sadselia S., Watkins K., Evans M., Ebay L. T., Dolan E. L. (2016). A social capital perspective on the mentoring of undergraduate life science researchers: An empirical study of undergraduate-postgraduate-faculty triads. CBE–Life Sciences Education.

[B3-behavsci-16-00798] Almager I. L., Cumby S., Almakdash M. H. (2021). Developing human capital through instructional leadership: Learning to coach during principal preparation. Open Journal of Leadership.

[B4-behavsci-16-00798] Augustine-Shaw D., Reilly M. (2017). I am mentor, I am coach. The Learning Professional.

[B5-behavsci-16-00798] Bastian K. C., Drake T. A., Otte A. (2025). Interns to leaders? Assessing whether principal internship characteristics predict entry into the school administrator workforce. Journal of Research on Leadership Education.

[B6-behavsci-16-00798] Bloom G., Castagna C., Warren B. (2003). More than mentors: Principal coaching. Leadership.

[B7-behavsci-16-00798] Boon Z. S. L. (2022). Coaching: An approach for leadership development in the Singapore education system. International Journal of Mentoring and Coaching in Education.

[B8-behavsci-16-00798] Coleman J. S. (1988). Social capital in the creation of human capital. The American Journal of Sociology.

[B9-behavsci-16-00798] Collaborative for Academic, Social, and Emotional Learning [CASEL] (n.d.). Fundamentals of SEL.

[B10-behavsci-16-00798] Collins C., Murphy R., Brown M. (2025). The power of coaching in the professional learning and development of school leaders: An ecological framework and critical insights from a systematic review. Frontiers in Education.

[B11-behavsci-16-00798] Cosner S., De Voto C. (2023). Using leadership coaching to strengthen the developmental opportunity of the clinical experience for aspiring principals: The importance of brokering and third-party influence. Educational Administration Quarterly.

[B12-behavsci-16-00798] Cosner S., Walker L., Swanson J., Hebert M., Whalen S. P. (2018). Examining the architecture of leadership coaching: Considering developmental affordances from multifarious structuring. Journal of Educational Administration.

[B13-behavsci-16-00798] Creswell J. W., Creswell J. D. (2018). Research design: Qualitative, quantitative, and mixed methods approaches.

[B14-behavsci-16-00798] Creswell J. W., Poth C. N. (2018). Qualitative inquiry and research design: Choosing among five approaches.

[B15-behavsci-16-00798] Crow G. M., Whiteman R. S. (2016). Effective preparation program features: A literature review. Journal of Research on Leadership Education.

[B16-behavsci-16-00798] Darling-Hammond L., Wechsler M. E., Levin S., Leung-Gagné M., Tozer S. (2022). Developing effective principals: What kind of learning matters? *[Report]*.

[B17-behavsci-16-00798] Dolan D. M., Willson P. (2019). Triad mentoring model: Framing an academic-clinical partnership practicum. Journal of Nursing Education.

[B18-behavsci-16-00798] Drake T. A., Seaton L. E., Ivey L. (2023). Isolated or integrated? Tracing the principal mentor–intern relationship across an academic year. Education Sciences.

[B19-behavsci-16-00798] Flick U. (2018). The SAGE handbook for qualitative data collection.

[B20-behavsci-16-00798] Fusarelli B. C., Fusarelli L. D. (2023). What do excellent school leader preparation programs look like?. Phi Delta Kappan.

[B21-behavsci-16-00798] Fusarelli B. C., Militello M. (2012). Racing to the top with leaders in rural high poverty schools. Planning and Changing.

[B22-behavsci-16-00798] Gray J. A. (2018). Leadership coaching and mentoring: A research-based model for school partnerships. International Journal of Education Policy and Leadership.

[B23-behavsci-16-00798] Halliwell P. R., Mitchell R. J., Boyle B. (2023). Leadership effectiveness through coaching: Authentic and change-oriented leadership. PLoS ONE.

[B24-behavsci-16-00798] Heifetz R. A., Laurie D. L. (1997). The work of leadership. Harvard Business Review.

[B25-behavsci-16-00798] Huggins K. S., Klar H. W., Andreoli P. M. (2021). Facilitating leadership coach capacity for school leadership development: The intersection of structured community and experiential learning. Educational Administration Quarterly.

[B26-behavsci-16-00798] International Coaching Federation (2025). What is coaching?.

[B27-behavsci-16-00798] James-Ward C. (2013). The coaching experience of four novice principals. International Journal of Mentoring and Coaching in Education.

[B28-behavsci-16-00798] Kappler-Hewitt K., Von Dohlen H., Weiler J., Fusarelli B., Zwadyk B. (2020). Architecture of innovative internship coaching models within US principal preparation programs. International Journal of Mentoring and Coaching in Education.

[B29-behavsci-16-00798] Knowles M. S., Holton E. F., Swanson R. A. (2005). The adult learner: The definitive classic in adult education and human resource development.

[B30-behavsci-16-00798] Lewis T. E., Jones K. D. (2019). Increasing principal candidates’ self-efficacy through virtual coaching. Journal of Organizational and Educational Leadership.

[B31-behavsci-16-00798] Lochmiller C. R. (2024). Leadership coaching relationships: A qualitative examination of underlying factors. Journal of Educational Administration.

[B32-behavsci-16-00798] Master B. K., Schwartz H., Unlu F., Schweig J., Mariano L. T., Coe J., Wang E. L., Phillips B., Bergland T. (2022). Developing school leaders: Findings from a randomized control trial study of the executive development program and paired coaching. Educational Evaluation and Policy Analysis.

[B33-behavsci-16-00798] Maxwell J. A. (2013). Qualitative research design: An interactive approach.

[B34-behavsci-16-00798] Newcomb W. S., Ensley L., Seaton L. E., Newcomb W. S., Seaton L. E. (2026). Coaching as a feminist leadership development strategy for women leaders. Teaching educational leadership.

[B35-behavsci-16-00798] New Teacher Center (n.d.). Pushing into practice: Instructional coaching.

[B36-behavsci-16-00798] Patrick S. K., Rogers L. K., Goldring E., Neumerski C. M., Robinson V. (2021). Opening the black box of leadership coaching: An examination of coaching behaviors. Journal of Educational Administration.

[B37-behavsci-16-00798] Roberts M. B., Gonzalez M. (2023). Combining mentoring and coaching to support aspiring leaders’ development: Participants’ voices. International Journal of Educational Leadership Preparation.

[B38-behavsci-16-00798] Rogers L. K., VanGronigen B. A. (2023). Beyond preparation: State approaches to early career principal induction. Leadership and Policy in Schools.

[B39-behavsci-16-00798] Salavert R. (2015). Coaching: An apprenticeship approach for the 21st century. International Journal of Educational Leadership and Management.

[B40-behavsci-16-00798] Saldaña J. (2013). The coding manual for qualitative researchers.

[B41-behavsci-16-00798] Shoho A. R., Barnett B. G., Martinez P. (2012). Enhancing” OJT” internships with interactive coaching. Planning and Changing.

[B42-behavsci-16-00798] Snodgrass Rangel V. S., Drake T. A., Butcher K. A., Seaton L. E. (2024). A synthesis of research on principal internships. Review of Educational Research.

[B43-behavsci-16-00798] Stevenson I. (2017). What a question can accomplish. The Learning Professional.

[B44-behavsci-16-00798] Su-Keene E., DeMatthews D. (2022). ‘Savoring’ the joy: Reducing principal burnout and improving well-being through positive psychology interventions. The Clearing House.

[B45-behavsci-16-00798] Taylor J. E., Mangin M. M., Stoelinga S. R. (2008). Instructional coaching: The state of the art. Effective teacher leadership: Using research to inform and reform.

[B46-behavsci-16-00798] Thessin R. A., Clayton J. K., Jamison K. (2020). Profiles of the administrative internship: The mentor/intern partnership in facilitating leadership experiences. Journal of Research on Leadership Education.

[B47-behavsci-16-00798] Thomas C., Koehn J., Turner J. (2024). Promising practices for leadership development: Exploring a collaborative professional learning and coaching program. Canadian Journal of Educational Administration and Policy.

[B48-behavsci-16-00798] Uen J. F., Chang H. C., McConville D., Tsai S. C. (2018). Supervisory mentoring and newcomer innovation performance in the hospitality industry. International Journal of Hospitality Management.

[B49-behavsci-16-00798] van Nieuwerburgh C., Barr M., Munro C., Noon H., Arifin D. (2020). Experiences of aspiring school principals receiving coaching as part of a leadership development programme. International Journal of Mentoring and Coaching in Education.

[B50-behavsci-16-00798] Watling C. J., LaDonna K. A. (2019). Where philosophy meets culture: Exploring how coaches conceptualize their roles. Medical Education.

[B51-behavsci-16-00798] Wise D., Cavazos B. (2017). Leadership coaching for principals: A national study. Mentoring & Tutoring: Partnership in Learning.

[B52-behavsci-16-00798] Wise D., Hammack M. (2011). Leadership coaching: Coaching competencies and best practices. Journal of School Leadership.

[B53-behavsci-16-00798] Yin R. K. (2016). Qualitative research from start to finish.

